# Impact of extracorporeal CPR with transcatheter heart pump support (ECPELLA) on improvement of short-term survival and neurological outcome in patients with refractory cardiac arrest – A single-site retrospective cohort study

**DOI:** 10.1016/j.resplu.2022.100244

**Published:** 2022-05-20

**Authors:** Takashi Unoki, Motoko Kamentani, Tomoko Nakayama, Yudai Tamura, Yutaka Konami, Hiroto Suzuyama, Masayuki Inoue, Megumi Yamamuro, Eiji Taguchi, Tadashi Sawamura, Koichi Nakao, Tomohiro Sakamoto

**Affiliations:** Department of Cardiology and Intensive Care Unit, Saiseikai Kumamoto Hospital, 5-3-1 Chikami, Minami-ku, Kumamoto City, Kumamoto 861-4193, Japan

**Keywords:** Cardiac arrest, Circulatory support, Extracorporeal cardiopulmonary resuscitation, Impella, LV unloading, Myocardial protection, ANOVA, Analysis of variance, CI, Confidential interval, CPC, Cerebral Performance Categories, CPR, Cardiopulmonary resuscitation, CVP, Central venous pressure, ECPELLA, Combination of VA-ECMO and Impella transcatheter heart pump support, E-CPR, Extracorporeal cardiopulmonary resuscitation, IABP, Intra-aortic balloon pump, LV, Left ventricle, MCS, Mechanical circulatory support, OHCA, Out-of-hospital cardiac arrest, PAPI, Pulmonary artery pulsatility index, VA-ECMO, Venoarterial extracorporeal membrane oxygenation, VIS, Vasoactive inotrope score

## Abstract

**Aim:**

Extracorporeal cardiopulmonary resuscitation (E-CPR) using veno-arterial extracorporeal membrane oxygenation (VA-ECMO) is a novel lifesaving method for refractory cardiac arrest. Although VA-ECMO preserves end-organ perfusion, it may affect left ventricular (LV) recovery due to increased LV load. An emerging treatment modality, ECPELLA, which combines VA-ECMO and a transcatheter heart pump, Impella, can simultaneously provide circulatory support and LV unloading. In this single-site cohort study, we assessed impact of ECPELLA support on clinical outcomes of refractory cardiac arrest patients.

**Method:**

We retrospectively reviewed 165 consecutive cardiac arrest patients, who underwent E-CPR by VA-ECMO with or without intra-aortic balloon pump (IABP) or ECPELLA from January 2012 to September 2021. We assessed 30-day survival rate, neurological outcome, hemodynamic data, and safety profiles including hemolysis, acute kidney injury, blood transfusion and embolic cerebral infarction.

**Results:**

Among 165 E-CPR patients, 35 patients were supported by ECPELLA, and 130 patients were supported by conventional VA-ECMO with or without IABP. Following propensity score matching of 30 ECPELLA and 30 VA-ECMO patients, the 30-day survival (ECPELLA: 53%, VA-ECMO: 20%, p < 0.01) and favorable neurological outcome determined by the Cerebral Performance Category score 1 or 2 (ECPELLA: 33%, VA-ECMO: 7%, p < 0.01) were significantly higher with ECPELLA. Patients receiving ECPELLA also showed significantly higher total mechanical circulatory support flow and lower arterial pulse pressure for the first 3 days (p < 0.01) of treatment. There were no statistical differences in safety profiles between treatment groups.

**Conclusion:**

ECPELLA may be associated with improved 30-day survival and neurological outcome in patients with refractory cardiac arrest.

## Introduction

Management of patients with refractory cardiac arrest who do not respond to conventional cardiopulmonary resuscitation (CPR) is controversial. Despite recent advances in the use of venoarterial extracorporeal membrane oxygenation (VA-ECMO) during extracorporeal cardiopulmonary resuscitation (E-CPR) by which oxygenated blood is supplied via the femoral artery, patient outcomes have not significantly improved.1., 2., 3., 4. It has been shown that earlier E-CPR improves neurological outcome.^5^ Although VA-ECMO can preserve end-organ perfusion with oxygenated blood, arterial blood perfusion by VA-ECMO increases injured left ventricular (LV) afterload. This increases myocardial wall tension by LV chamber distension and may lead to further myocardial damage.2., 5., 6..

Intra-aortic balloon pump (IABP) is often used as additive mechanical circulatory support (MCS) for treatment of patients in combination with VA-ECMO.7., 8. While IABP can reduce systolic LV afterload, the injured LV must eject blood into the systemic arterial tree to maintain end-organ perfusion. The LV afterload reduction by IABP appears to be quite limited due to significantly increased LV afterload by VA-ECMO.^6^ In addition, the arterial cannula placed at the distal side of the balloon during the diastolic phase can also interfere with the oxygenated blood supply. A recent report showed that the combined use of VA-ECMO and IABP for patients with cardiogenic shock did not improve the outcome compared to VA-ECMO alone.^9^ Therefore, it is critical to develop an alternative MCS modality in order to improve clinical outcomes of patients with refractory cardiac arrest.

A number of studies have recently shown that combined MCS using a transcatheter heart pump (Impella, Abiomed Inc. Danvers, MA, USA) and VA-ECMO for patients with refractory cardiogenic shock could improve short-term survival.10., 11., 12., 13., 14. The Impella pumps blood directly from the LV cavity and ejects blood in an antegrade direction to the ascending aorta to achieve simultaneous circulatory support and LV preload reduction.^15^ When it is combined with VA-ECMO, Impella not only contributes to circulatory support, but also significantly reduces LV load. Thus, considering these unique hemodynamic effects, the combination of Impella and VA-ECMO (ECPELLA), may confer superior clinical outcomes.

In our institute, we have been utilizing E-CPR with VA-ECMO for patients with refractory cardiac arrest to restore systemic circulation. Before September 2018, IABP was the only available adjunct MCS for cardiac arrest patients requiring E-CPR, but has been replaced by Impella in the majority of cases. In the present study, we retrospectively reviewed and assessed impact of ECPELLA treatment on 30-day survival and neurological outcome compared to conventional VA-ECMO with or without IABP treatment in refractory cardiac arrest patients who underwent E-CPR.

## Methods

The current single-center observational study was approved by the local Institutional Ethics Committee (Saiseikai Kumamoto Hospital, Approval: No. 875) and the study follows the Declaration of Helsinki.

### Patients

We retrospectively reviewed individual patient records from January 2012 to September 2021 of 165 consecutive refractory cardiac arrest patients who underwent E-CPR using VA-ECMO support. Our institutional E-CPR criteria included 1) collapse witnessed by a bystander or reliable report of estimated collapse time; 2) assumed cardiac origin of events; and 3) refractory ventricular arrhythmias or pulseless electric activity with short duration of cardiac arrest that could not be recovered by conventional CPR. Exclusion criteria included 1) apparent aortic dissection prior to the E-CPR; 2) non-cardiac origins including severe trauma and/or stroke; and 3) known poor prognosis or terminal malignancies.16., 17.

### Mechanical circulatory support devices

The MCS devices used in our institute were Terumo VA-ECMO system (CAPIOX, Terumo, Tokyo, Japan), MERA Unified VA-ECMO system (UNIMO, Senko Medical Trading Co. Tokyo, Japan), Getinge IABP system (Datascope CS100/CS300 or Cardiosave IABP Hybrid, Getinge Japan, Tokyo, Japan), and Impella 2.5 (until July 2019) or Impella CP (after August 2019).

### Adjunctive MCS

Additional MCS, i.e., IABP or Impella, was added by the primary physician’s decision at establishment of E-CPR and coronary artery angiography. Since Impella pump was available, Impella was mainly selected as the adjunctive MCS, except cases in which Impella could not be implanted (e.g., severe aortic valve disease).

### Management of MCS

*VA-ECMO with or without IABP*. VA-ECMO flow was maintained to achieve mean arterial pressure ≥ 65 mmHg on 2.5-3.0 L/min while maintaining systolic aortic valve opening (confirmed by echocardiography). In most cases, vasopressor and/or inotropes, such as noradrenaline or dobutamine, were used to maintain the arterial pressure and the aortic valve opening (blood ejection from the LV).

*ECPELLA*. Following the availability of Impella at our institute, VA-ECMO flow was adjusted to a perfusion index over 2.2 L/min/m^2^ to maintain sufficient end-organ perfusion. Simultaneous use of Impella was implemented to prevent the aortic valve opening achieving LV uncoupling (total LV support) since Impella pumps out the blood from the LV independent of LV contraction.^15^

### Patient management and cessation of treatment

Patients were then transferred to the intensive care unit, and hemodynamic status was continuously monitored using a pulmonary artery catheter; arterial oxygen saturation using SpO_2_ (measured on the right-hand finger); and/or regional cerebral saturation levels (rSO_2_, during ECPELLA support only).^18^ The central body temperature was maintained between 34 °C and 36 °C by ECMO circuit heat exchanger for the first 24 hours and then gradually returned to 37 °C for the next 8 to 24 hours.^19^ Weaning of VA-ECMO was initiated when serum lactate levels being returned within a normal range. After the VA-ECMO weaning, IABP or Impella was explanted when the patient’s own cardiovascular function was recovered.

Withdrawal of treatment was decided when 1) brain computed tomography (CT) examination revealed severe brain edema and brain death diagnosis; or 2) clinically pulseless electric activity or asystole was confirmed despite treatment.

### Endpoints

The primary endpoint was 30-day survival after initiation of E-CPR using VA-ECMO with or without IABP, or ECPELLA. The secondary endpoints included success rate of VA-ECMO weaning, rates of favorable neurological outcome defined by a Cerebral Performance Category (CPC) score of 1 or 2 at the timing of hospital discharge, renal replacement therapy (including continuous hemodiafiltration, and/or hemodialysis for newly developed acute kidney injury), hemolysis, and blood transfusion. Other data analyses included changes in heart rate, total MCS flow (total VA-ECMO flow and Impella flow), arterial pressure, pulmonary artery pressure, mean central venous pressure, and aortic pulse pressure. Pulmonary artery pulsatility index (PAPI) from MCS support day 1 to day 3, serum lactate levels from E-CPR initiation (VA-ECMO) to MCS day 3, and vasoactive-inotrope scores (VISs) from MCS support day 1 to day 3 were also included. The VIS was calculated as dopamine (μg/kg/min) + dobutamine (μg/kg/min) + 100 × epinephrine (μg/kg/min) + 10 × milrinone (μg/kg/min) + 10000 × vasopressin (unit/kg/min) + 100 × norepinephrine (μg/kg/min).

### Propensity score matching

The clinical background of refractory cardiac arrest patients is heterogeneous, and the timing of E-CPR also depends on patient arrival to the institute and the institutional E-CRP criteria. Therefore, we applied propensity score analysis with 1:1 score matching using dependent variables of age, sex, witnessed cardiac arrest, bystander CPR, out-of-hospital cardiac arrest (OHCA), shockable rhythm, acute coronary syndrome, and collapse to VA-ECMO time.^20^

### Statistical analysis

**Fig. 1:**
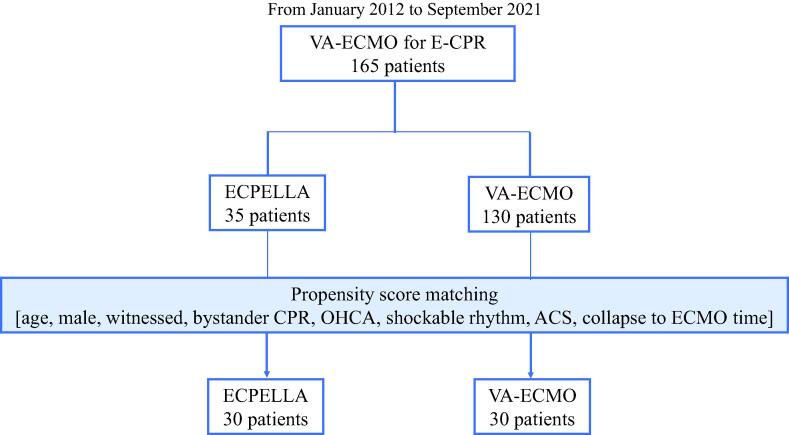
Patient enrollment flow chart. ACS: acute coronary syndrome, CPR: cardiopulmonary resuscitation, E-CPR: extracorporeal CPR, ECPELLA: Impella + VA-ECMO support, OHCA: out-of-hospital cardiac arrest, VA-ECMO: venoarterial extracorporeal membrane oxygenation.

All statistical analyses were conducted using JMP version 16.0 software. Comparison of concomitant IABP use, VA-ECMO weaning rate, favorable neurological outcome rate, and hemodynamic data was carried out by an extended Fisher’s exact test. Continuous variables were assessed by Student’s *t*-test (age), Wilcoxon test (creatinine and transfusion doses), and 1-way ANOVA with a Bonferroni post-hoc test (serum lactate, VIS, MCS flow, and hemodynamic data). When normalization for paired continuous data failed, we performed a Friedman test with Wilcoxon signed rank test for pairwise comparison. Kaplan-Meier survival curve analysis was conducted with log-rank test. The propensity score matching using a nearest neighbor approach with a caliper value at 0.2 (Supplemental Table 1 and Fig. 1).^21^ Statistical significance for all analyses was defined as p-value < 0.05.

## Results

### Patient characteristics

Among 165 refractory cardiac arrest patients, 35 patients received ECPELLA and 130 patients received VA-ECMO in which 48 patients received single VA-ECMO support and 82 patients received VA-ECMO with IABP support (Fig. 1, Table 1). OHCA occurred 40% of patients that received ECPELLA and 32% of VA-ECMO, respectively (p = 0.35, Table 1). Patients with IABP support (63% of patients before propensity score matching and 73% after matching, Table 1) within the VA-ECMO group showed no statistically significant difference in 30-day survival rate compared to patients with single VA-ECMO support after propensity score matching (VA-ECMO + IABP: 20.5%, VA-ECMO alone: 12.5%, p = 0.558). Patients that received ECPELLA support showed lower rate of pulseless electric activity (ECPELLA 40% vs. VA-ECMO 61%, p = 0.03), higher rate of acute coronary syndrome (66% vs. 45%, p < 0.01), and shorter collapse to VA-ECMO time (27 vs. 41 minutes, p < 0.01). Of note, no ECPELLA patients received concomitant IABP support after ECPELLA was established (Table 1).

**Table 1:** Patient characteristics.

	Prior to Propensity Score Matching			After Propensity Score Matching		
	ECPELLA (35)	VA-ECMO (130)	*p*-value	ECPELLA (30)	VA-ECMO (30)	*p*-value
Age, years	64 ± 14	67 ± 15	0.30	67 ± 12	67 ± 16	0.86
Male	25 (71)	85 (65)	0.50	22 (73)	19 (63)	0.40
Witness	33 (94)	120 (92)	0.68	29 (97)	28 (93)	0.55
Bystander-CPR,	30 (86)	116 (89)	0.57	27 (90)	27 (90)	1.00
Initial rhythm						
Shockable rhythm*	17 (49)	42 (32)	0.08	13 (43)	13 (43)	1.00
PEA	14 (40)	79 (61)	0.03	13 (43)	16 (53)	0.44
Asystole	4 (11)	9 (7)	0.40	4 (13)	1 (3)	0.15
OHCA	14 (40)	41 (32)	0.35	10 (33)	6 (20)	0.24
Serum creatinine, *mg/dL*	1.05 [0.85–1.52]	1.16 [0.89–1.50]	0.67	1.07 [0.85–1.56]	1.22 [0.97–1.52]	0.55
History of HD	2 (6)	4 (3)	0.48	2 (7)	1 (3)	0.55
Acute coronary syndrome	23 (66)	59 (45)	< 0.01	21 (70)	20 (67)	0.72
Collapse to VA-ECMO time, min	27 [13–44]	41 [24–64]	< 0.01	32 [19–46]	26.5 [16–38]	
Concomitant use of IABP	0 (0)	82 (63)	< 0.01	0 (0)	22 (73)	< 0.01

Data were expressed as n (%), mean ± standard deviation or median [inter-quartile range]. Statistical significance was defined as p < 0.05. ACS: acute coronary syndrome, CPR: cardiopulmonary resuscitation, VA-ECMO: venoarterial extracorporeal membrane oxygenation, ECPELLA: VA-ECMO + Impella, HD: hemodialysis, OHCA: out-of-hospital cardiac arrest, PEA: pulseless electric activity.

* Included pulseless ventricular tachycardia and ventricular fibrillation.

For propensity score analysis, 8 explanatory variables described in the Methods section were applied. Table 1 summarizes clinical characteristics of the patients after the propensity score matching in which 30 patients in ECPELLA group and 30 patients in VA-ECMO group were included for further analyses.

### Outcomes

**Fig. 2:**
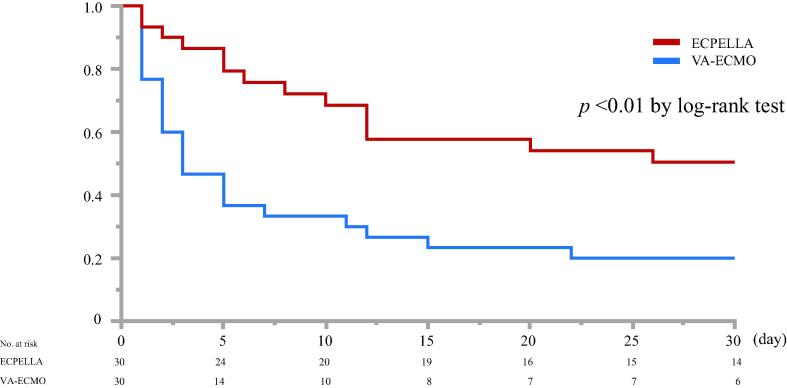
Kaplan-Meier survival curve analysis and 30-day survival rate. 30-day survival of ECPELLA patients compared to patients receiving VA-ECMO (*p* < 0.01).

The rate of successful VA-ECMO weaning with ECPELLA was significantly higher than patients receiving VA-ECMO (63% vs. 27%, p < 0.01). Favorable neurological outcome was also significantly higher with ECPELLA compared to VA-ECMO (33% vs. 7%, p < 0.01) (Table 2). Kaplan-Meier survival analysis revealed that ECPELLA resulted in a significantly higher 30-day survival rate compared to VA-ECMO (53% vs. 20%, p < 0.01 by log-rank test, Fig. 2, Table 2).

**Table 2:** ECMO weaning rate, neurological outcome, and hemodynamic data.

	ECPELLA (30)	VA-ECMO (30)	*p*-value
VA-ECMO weaning	19 (63)	8 (27)	< 0.01
Favorable neurological outcome*	10 (33)	2 (7)	< 0.01
30-day survival	16 (53)	6 (20)	< 0.01
Hemodynamic Data			
D1-heart rate*, bpm*	76 ± 22	79 ± 23	0.59
D2-heart rate*, bpm*	66 ± 20	78 ± 28	< 0.01
D3-heart rate*, bpm*	68 ± 22	83 ± 29	0.09
D1-MAP*, mmHg*	75 ± 15	60 ± 20	< 0.01
D2-MAP*, mmHg*	72 ± 17	69 ± 25	0.63
D3-MAP*, mmHg*	67 ± 14	59 ± 16	0.08
D1-Arterial pulse pressure*, mmHg*	11 [8–33]	34 [19–55]	< 0.01
D2-Arterial pulse pressure*, mmHg*	13 [4.5–34]	43 [20–72]	< 0.01
D3-Arterial pulse pressure*, mmHg*	17.5 [6–36]	40 [23–68]	< 0.01
D1-mPAP*, mmHg*	16 ± 5	18 ± 9	0.29
D2-mPAP*, mmHg*	20 ± 7	21 ± 11	0.75
D3-mPAP*, mmHg*	17 ± 5	20 ± 7	0.21
D1-PAPI	1.3 [1.1–3.6]	3.8 [2.1–6.0]	< 0.01
D2-PAPI	4.3 [0–11.2]	3.1 [0–10.1]	0.96
D3-PAPI	5.0 [0–10.3]	5.9 [0–17.3]	0.36
D1-CVP*, mmHg*	9.5 ± 4.8	11.4 ± 6.8	0.29
D2-CVP*, mmHg*	11.2 ± 3.7	13.5 ± 7.1	0.18
D3-CVP*, mmHg*	9.4 ± 3.7	14.3 ± 6.4	< 0.05
D1-Vasoactive-inotrope score	4.3 [0–11.2]	3.1 [0–10.1]	0.96
D2-Vasoactive-inotrope score	5.0 [0–10.3]	5.9 [0–17.3]	0.36
D3-Vasoactive-inotrope score	1.0 [0–7.8]	11.1 [0–18.7]	0.07

Data were expressed as n (%), mean ± standard deviation or median [inter-quartile range]. Statistical significance was defined as p < 0.05. CVP: central venous pressure, D1, D2, D3 are, respectively, mechanical circulatory support day 1, day 2, and day 3. ECMO: extracorporeal membrane oxygenation, ECPELLA: venoarterial-ECMO + Impella, MAP: mean arterial pressure, OHCA: out-of-hospital cardiac arrest, PAPI: pulmonary artery pulsatile index.

* defined as cerebral performance category (CPC) scales at 1 or 2 at the timing of hospital discharge.

**Fig. 3:**
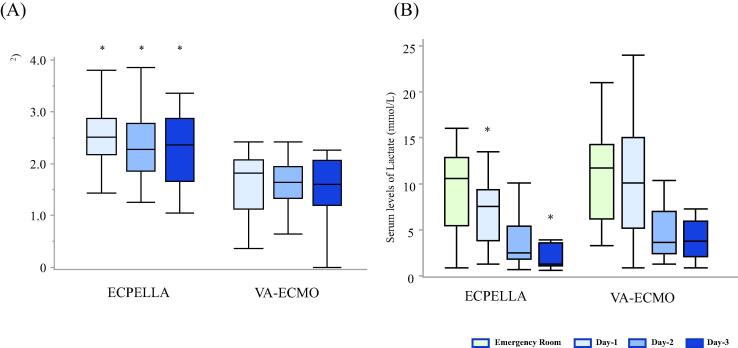
**Total mechanical circulatory support (MCS) flow, and serum lactate levels.** Panel A: Total MCS flows (*: p < 0.01 vs. VA-ECMO by Wilcoxon test); Panel B: Serum lactate levels with ECPELLA and VA-ECMO on day1 and day 3 (p < 0.01).

Total MCS flows with ECPELLA were significantly higher from MCS day 1 to day 3 (p < 0.01, Fig. 3A). While serum lactate levels with ECPELLA were significantly decreased from the emergency room arrival to MCS day 3 (Emergency Room:10.6 [5.4–12.5], day-1: 7.0 [3.8–9.1], day-2: 2.4 [1.7–4.5], and day-3: 1.6 [1.1–2.4], median [IQR] mmol/L), VA-ECMO resulted in significantly decreased lactate levels only on MCS day 2 and day 3 (Emergency Room:11.5 [7.6–14.3], day-1: 9.2 [5.7–12.7], day-2: 3.3 [1.9–4.8], and day-3: 2.7 [1.6–5.4], Fig. 3B). Of note, serum lactate levels in patients with ECPELLA at day1 and day 3 were significantly lower than in patients receiving VA-ECMO on the same MCS support days (p < 0.01 vs. VA-ECMO, Fig. 3B).

The heart rate of patients in the ECPELLA group was significantly lower on MCS day 2 (p < 0.01, Table 2). The mean arterial pressure of ECPELLA patients on MCS day 1 was significantly higher than those receiving VA-ECMO (p < 0.01, Table 2). The mean pulmonary arterial pressure was similar between groups, while the arterial pulse pressure from MCS day 1 to day 3, and PAPI at day-1 were significantly lower with ECPELLA (p < 0.01, Table 2). Mean central venous pressure (CVP) in ECPELLA patients was also lower on MCS day 3 (p < 0.01, Table 2). There were no significant differences in VISs from MCS day 1 to day 3 regardless of treatment groups (Table 2).

### Safety profiles

Table 3 shows safety-related parameters. Hemolysis was reported 33% of ECPELLA patients and 16% VA-ECMO (p = 0.13). Newly developed acute kidney injury requiring renal replacement therapy including continuous hemodiafiltration and/or hemodialysis was reported in 7% of ECPELLA patients and 20% of VA-ECMO patients, respectively (p = 0.12). The number of patients who required blood transfusion (including red blood cell, fresh frozen plasma, and platelets) was similar in both treatment groups (98% ECPELLA and 93% VA-ECMO). There were no differences in blood transfusion amounts between treatment groups. Rates of embolic cerebral infarction (p = 0.19) were not statistically significant between groups. Finally, while twice as many embolic cerebral infarction occurred in patients receiving ECPELLA (n = 8) compared to VA-ECMO (n = 4). This failed to reach statistical significance.

**Table 3:** Safety related parameters.

	ECPELLA (30)	ECMO (30)	*p-value*
Hemolysis	10 (33)	5 (16)	0.13
AKI required RRT*	2 (7)	6 (20)	0.12
Blood transfusion	29 (97)	28 (93)	0.55
RBC transfusion,	29 (97)	27 (90)	0.29
Amount of RBC, *unit*	19 [11–27]	18 [6–46]	0.88
Amount of FFP, *unit*	16 [8–20]	14 [0–38]	0.78
Amount of Platelet, *unit*	10 [0–30]	0 [0–33]	1.00
Embolic cerebral infarction	8 (27)	4 (13)	0.19

Data were expressed as n (%) or median [IQR]. AKI: acute kidney injury, FFP: fresh frozen plasma, RBC: red blood cell, RRT: renal replacement therapy. Statistical significance was defined as p < 0.05.

* Included continuous hemodiafiltration and/or hemodialysis.

## Discussion

Beneficial effect of ECPELLA on E-CPR patients has been recently reported by Gaisendrees et al. in which ECPELLA significantly reduced all-cause mortality and improved VA-ECMO weaning rate.^13^ In the present study, we found that ECPELLA support was associated with higher rate of VA-ECMO weaning and 30-day survival similar to the previous study.^13^ Patients received ECPELLA support exhibited higher mean arterial pressure on MCS day 1; lower pulse pressure from MCS days 1 to 3; and a lower heart rate on MCS day 2. However, VIS did not show difference between ECPELLA and VA-ECMO. These results suggest that ECPELLA maintains arterial perfusion pressure and systemic perfusion flow without additional vasopressors and/or inotropes.

Gaisendrees et al. also reported that rate of acute kidney injury requiring renal replacement therapy of ECPELLA support was higher compared to single VA-ECMO support.^13^ In the current study, the incidence rate of acute kidney injury did not show statistical difference between ECPELLA and VA-ECMO. We speculate the difference between our results and their results could be due to ECPELLA initiation timing and higher total circulatory support achieved by ECPELLA in our institute.13., 22. Gaisendrees et al., described that ECPELLA timing was determined by levels of LV distension, blood retention within the LV cavity (spontaneous contraction by echocardiography), and pulmonary edema due to increased afterload by VA-ECMO.^13^ In contract, we established ECPELLA support at the earliest time point immediately after the establishment of E-CPR to obtain a VA-ECMO perfusion index ≥ 2.2 L/min/m^2^ preventing the aortic valve opening in which Impella was used to directly pump out the blood from the LV cavity increasing total MCS flow (total LV support)^15^ Further studies are necessary whether reduction of acute kidney injury is associated with ECPELLA support timing, higher total circulatory support flow, and/or arterial perfusion pressure.

In the present study, we also found ECPELLA displayed lower PAPI on MCS day 1 and CVP on MCS day 3 without elevation of mean pulmonary arterial pressure suggesting that ECPELLA reduced the right ventricular preload. We have previously reported a case of increased coronary arterial flow by LV uncoupling (total LV support) by ECPELLA in which reduced arterial pulse pressure appeared to be associated with total LV support.^23^ These results imply that superior clinical outcomes might be associated with both right (lower PAPI and CVP) and left ventricular unloading effects to reduce myocardial damage with higher total MCS flow compared to VA-ECMO. Saku at el. reported that LV uncoupling (total LV support) achieved by Impella was significantly reduced infarct size and prevents subsequent heart failure in a dog model.^15^ Previous reports also showed that VA-ECMO dramatically increased LV load and additional IABP support was not enough to reduce the increased LV load by VA-ECMO. In contrast, ECPELLA could significantly reduce LV load compared to VA-ECMO or VA-ECMO with IABP support suggesting that ECPELLA has higher myocardial protection effect.24., 25., 26. Taken together, further studies are necessary to investigate the impact of ECPELLA support on myocardial protection effect.

It is well-known that VA-ECMO can directly supply oxygenated blood throughout systemic circulation during E-CPR. Previous studies showed that E-CPR has a significant beneficial effect on both early patient survival and favorable neurological outcome.27., 28. In the current study, the rate of CPC 1 or 2 with ECPELLA was 33% and 7% with VA-ECMO. In contrast, the incidence of embolic cerebral infarction of ECPELLA patients did not significantly differ compared to VA-ECMO and only survivors showed improved neurological outcome. Further studies are also necessary to confirm the effects of ECPELLA on favorable neurological outcome.

There are several limitations of the current study. First, this is a single center, retrospective cohort in which historical clinical experience in the institute must be considered when comparing the treatment modalities since the Impella became available in 2018 for E-CPR. Therefore, we could not adjust/allocate treatment modalities like a randomized or case-control studies. Second, since the cardiac arrest patient population presents heterogeneously, we could not exclude other potential confounding factors from the current dataset. Therefore, prospective randomized studies are necessary to evaluate ECPELLA effects compared to conventional VA-ECMO. However, enrolling sufficient patients to a randomized study is difficult in this patient population due to both ethical and practical reasons. Third, although we did not find statistical differences in major adverse events between ECPELLA and VA-ECMO groups, the number of patients who developed hemolysis (p = 0.13), acute kidney injury (p = 0.12) and embolic cerebral infarction (p = 0.19) appeared to be higher in ECPELLA group. This could be statistically underpowered and therefore unable to detect differences due to relatively small sample numbers.

## Conclusions

In this retrospective cohort study using propensity score matching, our results suggest that ECPELLA was associated with improved mortality and favorable neurological outcome in patients with cardiac arrest who underwent E-CPR. Further studies including multicenter observational studies are necessary to determine whether ECPELLA can be the first-choice therapeutic option for patients with cardiac arrest.
