# Supplementary material for: Impact of extracorporeal CPR with transcatheter heart pump support (ECPELLA) on improvement of short-term survival and neurological outcome in patients with refractory cardiac arrest – A single-site retrospective cohort study
Source: Resusc Plus. 2022 May 20;10:100244. doi: 10.1016/j.resplu.2022.100244 (PMC9127400; doi:10.1016/j.resplu.2022.100244)
Supplement: Supplementary data 1 [file mmc1.docx]

Supplementary data Table 1. Nominal logistic regression analysis of dependent variables (Impella as an objective variable)

| Variable | Likelihood Ratio (Chi sq) | p-value |
| --- | --- | --- |
| Out-of-hospital cardiac arrest | 4.06 | <0.05 |
| Collapse to ECMO time | 18.23 | <0.05 |
| Acute Coronary Syndrome | 4.52 | <0.05 |
| Age | 0.56 | 0.46 |
| Shockable Rhythm | 2.00 | 0.16 |
| Male | 0.17 | 0.68 |
| Bystander CPR | 0.02 | 0.88 |
| Witness | 0.02 | 0.90 |

For propensity score analysis, 8 explanatory variables were selected. Rates of out-of-hospital cardiac arrest, collapse to ECMO time, and acute coronary artery showed significantly difference between ECPELLA and ECMO groups. The concordance index calculated by area under the curve of receiver-operator characteristic (ROC) curve analysis was 0.752 (Supplemental Figure 1A).

Supplementary Table 2. Propensity score matching results

|  | Pre-propensity score matching | | Post-propensity score matching | |
| --- | --- | --- | --- | --- |
|  | ECPELLA | ECMO | ECPELLA | ECMO |
| Number | 35 | 130 | 30 | 30 |
| Mean | -0.78 | -1.90 | -1.06 | -1.06 |
| Standard Deviation | 1.12 | 1.18 | 0.90 | 0.90 |

Propensity score analysis using a 1:1 nearest neighbor matching with caliper coefficient at 0.2 was carried out. There were 30 ECPELLA and 30 ECMO cases were matched with caliper value at 0.229 by which 5 ECPELLA cases were rejected by the propensity score matching.

**Supplementary Figure**

**
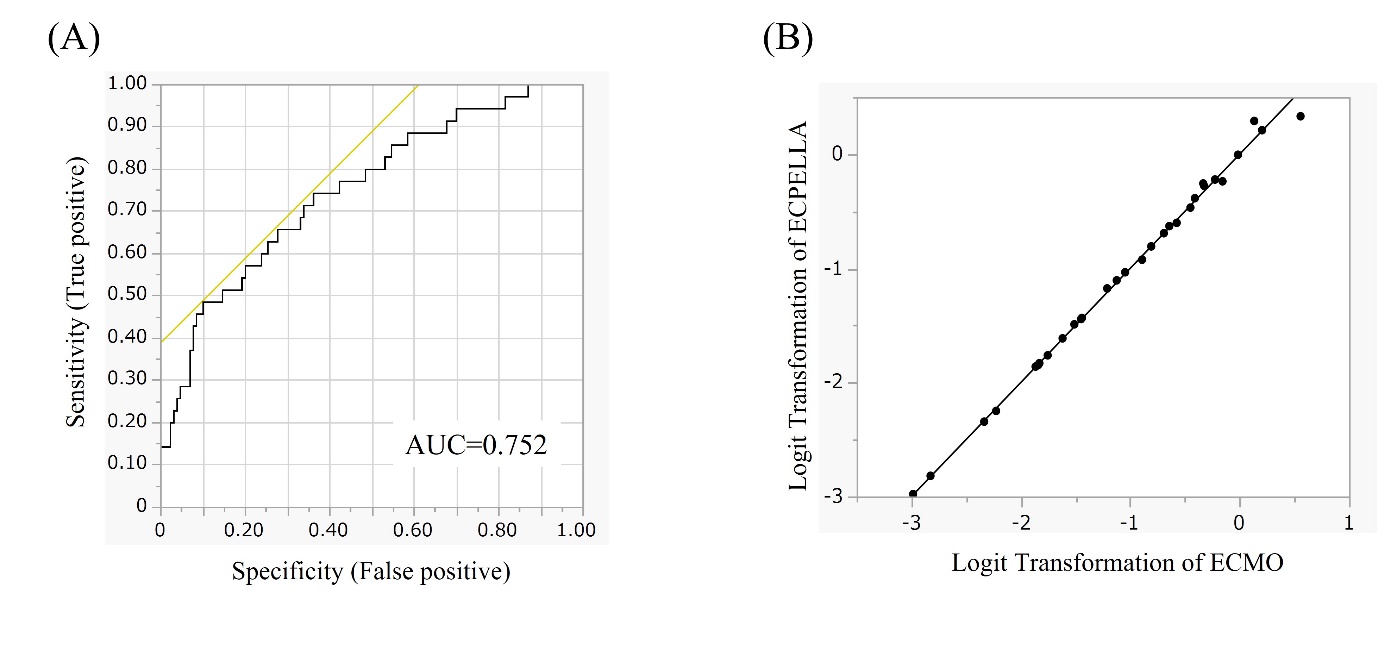
**

Propensity Score Analysis. (A) Receiver operating characteristic (ROC) curve analysis of the propensity scores. The AUC was 0.752 indicating propensity scores were valid on the logistic regression model. (B) Logit transformation results of post-propensity matching variables indicating the propensity matching was acceptable.
